# The Effect of Lysophosphatidic Acid during *In Vitro* Maturation of Bovine Oocytes: Embryonic Development and mRNA Abundances of Genes Involved in Apoptosis and Oocyte Competence

**DOI:** 10.1155/2014/670670

**Published:** 2014-03-04

**Authors:** Dorota Boruszewska, Ana Catarina Torres, Ilona Kowalczyk-Zieba, Patricia Diniz, Mariana Batista, Luis Lopes-da-Costa, Izabela Woclawek-Potocka

**Affiliations:** ^1^Department of Reproductive Immunology and Pathology, Institute of Animal Reproduction and Food Research, Polish Academy of Sciences, 10-747 Olsztyn, Poland; ^2^CIISA, Faculty of Veterinary Medicine, University of Lisbon, 1300-477 Lisbon, Portugal

## Abstract

In the present study we examined whether LPA can be synthesized and act during *in vitro* maturation of bovine cumulus oocyte complexes (COCs). We found transcription of genes coding for enzymes of LPA synthesis pathway (*ATX* and *PLA2*) and of LPA receptors (*LPAR 1–4*) in bovine oocytes and cumulus cells, following *in vitro* maturation. COCs were matured *in vitro* in presence or absence of LPA (10^−5^ M) for 24 h. Supplementation of maturation medium with LPA increased mRNA abundance of *FST* and *GDF9* in oocytes and decreased mRNA abundance of *CTSs* in cumulus cells. Additionally, oocytes stimulated with LPA had higher transcription levels of *BCL2* and lower transcription levels of *BAX* resulting in the significantly lower *BAX/BCL2* ratio. Blastocyst rates on day 7 were similar in the control and the LPA-stimulated COCs. Our study demonstrates for the first time that bovine COCs are a potential source and target of LPA action. We postulate that LPA exerts an autocrine and/or paracrine signaling, through several LPARs, between the oocyte and cumulus cells. LPA supplementation of maturation medium improves COC quality, and although this was not translated into an enhanced *in vitro* development until the blastocyst stage, improved oocyte competence may be relevant for subsequent *in vivo* survival.

## 1. Introduction 

During the last decade, the functional role of lysophosphatidic acid (LPA) in the female reproduction has been the object of an increasing number of reports [1]. LPA, the simplest and at the same time one of the most potent phospholipids, has been regarded as an important signaling molecule participating in various biological processes, such as cell proliferation [2], differentiation [3], survival [4, 5], morphogenesis [6], and cytokine secretion [7]. This molecule is produced from membrane phospholipids by two main pathways/enzymes: autotaxin (ATX) and phospholipase A2 (PLA2) [8, 9]. In mammals, LPA exerts its action *via* at least six high affinity, transmembrane G-protein-coupled receptor (GPCR) types: LPAR1–LPAR6 and possibly through a nuclear receptor PPAR*γ* [10–14]. Expression of LPARs is tissue and cell specific [15]. An association of LPA signaling with regulation of reproductive function first was described in women [16] and then in farm animals including ruminants [17, 18]. Our previous studies showed that LPA is locally produced and acts in the bovine uterus [18, 19] and ovary [20, 21]. We documented that the *intravaginal *administration of LPARs antagonist decreases pregnancy rate and that infusion of LPA prevents spontaneous luteolysis, prolongs the functional lifespan of the corpus luteum (CL), and also stimulates luteotropic prostaglandin (PG)E_2_ synthesis in heifers [18, 22]. Additionally, in *in vitro *studies, we found a stimulatory effect of LPA on progesterone (P_4_) synthesis and interferon (IFN)*τ* action in the steroidogenic cells of the bovine CL [20] and on luteotropic PGE_2_ synthesis in bovine endometrial cells [22]. Our recently published data demonstrates that bovine granulosa cells of the follicle are also the site of LPA synthesis and the target for LPA action [21]. In the above mentioned study [21], LPA exerted an autocrine and paracrine action in granulosa cells through several LPARs and stimulated estradiol (E_2_) synthesis *via* increased FSH receptor (*FSHR*) and 17**β** -hydroxysteroid dehydrogenase (*17*β*-HSD*) expression. These results led us to hypothesize that cumulus oocyte complexes (COCs) may be the source and the target of LPA action during oocyte maturation. Oocyte maturation involves changes in the nucleus [23–25] and cytoplasm [26–29], and granulosa cells modulate chromatin configuration [30, 31], transcriptional activity [31], and cytoplasmic maturation [30, 32]. This bidirectional communication between the oocyte and surrounding cumulus cells is essential for proper oocyte maturation and determinates subsequent oocyte competence.

There are two major premises that initiated our studies on the effect of LPA supplementation of maturation medium on the communication between the oocyte and surrounding cumulus cells defined as embryonic development and the expression of genes involved in apoptosis and oocyte competence in oocytes and cumulus cells. The first premise documented by Boruszewska et al. [21] is concerned about an autocrine and paracrine action of LPA in the granulosa cells of the bovine ovarian follicle. The second premise regards the continuous, unrestrained development of the methods of *in vitro* culture of bovine embryos using variously supplemented media.

In the present study we examined whether LPA can be synthesized and act during *in vitro* maturation of bovine COCs. We also determined the effect of LPA supplementation of maturation medium on mRNA abundance of oocyte quality markers (follistatin-*FST*, growth and differentiation factor 9-*GDF9*, bone morphogenetic protein 15-*BMP15*, cysteine proteinases-cathepsins: *CTSB, CTSK, CTSS,* and *CTSZ*) and genes involved in apoptosis (*BCL2* and *BAX*, as well as *BAX/BCL2* ratio) in oocytes and cumulus cells. Finally, we evaluated the effect of LPA supplementation of maturation medium on cleavage and blastocyst rates on day 2 and day 7 of *in vitro* culture of bovine embryos, respectively.

## 2. Materials and Methods

### 2.1. Chemicals and Suppliers

All chemicals and reagents for *in vitro *culture were purchased from Sigma Aldrich (Germany) unless otherwise stated. Plastic dishes, four-well plates, and tubes were obtained from Nunc (Thermo Scientific, Denmark). All chemicals for reverse transcription were acquired from Invitrogen (Life Technologies, USA).

### 2.2. Oocyte and Cumulus Cell Collection

All experimental procedures were approved by the Local Animal Care and Use Committee in Olsztyn, Poland (Agreement number 34/2012/N). Ovaries were collected from slaughtered cows and transported to the laboratory in sterile PBS at 37°C. COCs were obtained by aspiration from subordinate ovarian follicles, less than 5 mm in diameter. Only COCs consisting of oocytes with homogeneous ooplasm without dark spots and surrounded by at least three layers of compact cumulus cells were selected for the study. COCs were chosen under a stereomicroscope and washed two times in wash medium (TCM-199; #M2154) supplemented with 25 *μ*g/mL amphotericin b (#A2942), 5 USP/mL heparin (#H3393), 25 mM HEPES (#H3784), 5 mM sodium bicarbonate (#S4019), 0.2 mM sodium pyruvate (#P3662), and 1% fetal bovine serum (FBS; #12106C) and subsequently washed in maturation medium.

### 2.3. Oocyte Maturation

26 groups of 25 immature COCs were cultured in four-well plates (#144444) containing 400 *μ*L of maturation medium (TCM-199 supplemented with 0.4 mM L-glutamine (#G5763), 0.05 mg/mL gentamicin (#G1272), 1 *μ*L/mL insulin-transferrin-sodium selenite, (ITS, #I3146), 10 UI/mL pregnant mare's serum gonadotropin (PMSG), and 5 UI/mL chorionic gonadotropin human (hCG; PG600, Intervet International, Boxmeer, The Netherlands) and 15% *v/v* FBS) under 400 *μ*L of mineral oil (#M5310), as recently described by Torres et al. [33]. Two experimental groups were randomly generated for analyses from COCs: exposed to LPA agonist (LPA; 1-oleoyl-sn-glycerol 3-phosphate sodium salt; 10^−5^ M; #L7260) or PBS (control group) during *in vitro *maturation. The dose of LPA was taken from earlier reports on humans and rodents [34–36]. Subsequently COCs were matured *in vitro* for 24 h at 39°C under 5% CO_2_ in humidified air. After *in vitro* maturation, COCs were processed for total RNA extraction (for mRNA expression analysis) or *in vitro* fertilized and cultured (for cleavage and blastocyst rates analysis).

### 2.4. Sample Collection for RNA Isolation and Reverse Transcription

After 24 h of *in vitro* maturation, for total RNA extraction (for mRNA expression analysis), the oocytes from 5 pools of each experimental group (control or LPA treated) were separated from cumulus cells by vortexing. Each pool consisted of 25 denuded oocytes and all cumulus cells separated from the respective oocytes. The oocytes and cumulus cells were suspended in the Extraction Buffer and processed for RNA isolation according to manufacturer's instructions (#KIT0204, Arcturus PicoPure RNA Isolation Kit, Applied Biosystems, Life Technologies, USA). DNase treatment was performed for the removal of genomic DNA contamination using RNase-free DNase Set (#79254, Qiagen, Germany). Samples were stored at −80°C until reverse transcription. The reverse transcription (RT) was performed using oligo (dT)12–18 primers (#18418-012) by Super Script III reverse transcriptase (#18080-044) in a total volume of 20 *μ*L to prime the RT reaction and produce cDNA. The RT reaction was carried out at 65°C for 5 min and 42°C for 60 min followed by a denaturation step at 70°C for 15 min. RNase H (#18021-071) was used to degrade the RNA strand of an RNA-DNA hybrid (37°C for 20 min). RT products were diluted four times and were stored at −20°C until real-time PCR amplification.

### 2.5. Quantitative Real-Time PCR

The quantification of mRNA for the examined genes was conducted by real-time PCR using specific primers for *LPAR1, LPAR2, LPAR3, LPAR4, ATX, PLA2, FST, GDF9, BMP15, CTSB, CTSK, CTSS, CTSZ, BCL2, *and *BAX*. The results of mRNA expression were normalized to glyceraldehyde-3-phosphate dehydrogenase (*GAPDH*, an internal control) mRNA expression and were expressed as arbitrary units. The primers were designed using an online software package (http://bioinfo.ut.ee/primer3/). Primer sequences and the sizes of the amplified fragments of all transcripts are shown in Table 1. Real-time PCR was performed with an ABI Prism 7900 (Applied Biosystems, Life Technologies, USA) sequence detection system using Maxima SYBR Green/ROX qPCR Master Mix (#K0222, Fermentas, Thermo Scientific, USA). The PCR reactions were performed in 96-well plates. Each PCR reaction well (20 *μ*L) contained 2 *μ*L of RT product, 5 pmol/*μ*L forward and reverse primers each, and 10 *μ*L SYBR Green PCR master mix. In each reaction we used a quantity of cDNA equivalent to 0.25 oocyte or cumulus cells. Real-time PCR was performed under the following conditions: 95°C for 10 min, followed by 40 cycles of 94°C for 15 sec and 60°C for 60 sec. Subsequently in each PCR reaction melting curves were obtained to ensure single product amplification. In order to exclude the possibility of genomic DNA contamination in the RNA samples, the reactions were also performed either with blank-only buffer samples or in absence of the reverse transcriptase enzyme. The specificity of the PCR products for all examined genes was confirmed by gel electrophoresis and by sequencing. The efficiency range for the target and the internal control amplifications balance was between 95 and 100%. For the relative quantification of the mRNA expression levels real-time PCR Miner algorithm was used (http://www.miner.ewindup.info/version2).

### 2.6. *In Vitro* Fertilization and Embryo Culture

Procedures of *in vitro *fertilization and embryo culture were performed according to Torres et al. [33]. Briefly, for *in vitro* fertilization (for developmental capacity analysis), 16 pools of 25 COCs were washed in fertilization medium (modified Tyrode's medium (TALP) supplemented with 5.4 USP/mL heparin, 10 mM penicillamine (#P4875), 20 mM hypotaurine (#H1384), 0.25 mM epinephrine (#E1635), and 0.1 mg/mL gentamicin solution. For *in vitro* insemination, frozen-thawed semen was used. After thawing, semen was layered below capacitation medium (TALP medium supplemented with 72.72 mM pyruvic acid sodium pyruvate and 0.05 mg/mL gentamicin) and incubated for 1 h at 39°C in a 5% CO_2_ in humidified air atmosphere to allow the recovery of motile sperm through the swim-up procedure. After incubation, the upper two-thirds of the capacitation medium were recovered, centrifuged at 200 ×g for 10 min, the supernatant removed, and the sperm pellet diluted in an appropriate volume of fertilization medium to give a final concentration of 10^6^ sperm/mL. Groups of 25 COCs were coincubated with spermatozoa in four-well dishes containing 400 *μ*L of fertilization medium under 400 *μ*L of mineral oil for 48 h at 39°C in a 5% CO_2_ humidified air atmosphere. The day of *in vitro* insemination was considered day 0. At 48 h postinsemination (hpi) embryos were separated from cumulus cells by vortexing and washed three times in wash medium. The cleavage rates were assessed and embryos with four or more cells were placed in four-well dishes containing 400 *μ*L culture medium (SOF; synthetic oviductal fluid medium described by Holm et al. [37] supplemented with amino acids: 30 *μ*L/mL BME (#B6766) and 10 *μ*L/mL MEM (#M7145), 0.34 mM trisodium-citrate (#6448.1000, Merck Millipore, Germany), 2.77 mM myo-inositol (#I7508), 1 *μ*L/mL gentamicin, 1 *μ*L/mL ITS, and 5% *v/v* FCS) overlaid with 400 *μ*L mineral oil. Culture was carried out at 39°C in a 5% CO_2_ in air with high humidity. Blastocyst numbers were determined on day 7 postinsemination. The rates of development to the blastocyst stage were calculated based on the total number of matured oocytes.

### 2.7. Statistical Analysis

All data concerning expression patterns of target genes are presented as mean ± SEM. One-way ANOVA followed by Newman-Keuls' multiple comparison test was used to determine differences in mRNA expression of *LPARs* in oocytes and cumulus cells (GraphPad PRISM 6.0). Differences in transcription levels of the remaining genes were analyzed by Student's *t*-test for independent pairs. Cleavage and blastocyst rates were analyzed by Fisher's exact test. Differences were considered statistically significant at the 95% confidence level (*P* < 0.05).

## 3. Results

### 3.1. The Expression Patterns of *LPARs* (*LPAR1*, *LPAR2*, *LPAR3*, and *LPAR4*) and Enzymes Involved in LPA Synthesis (*ATX* and *PLA2*) in Oocytes and Cumulus Cells after *In Vitro* Maturation

**Figure 1:**
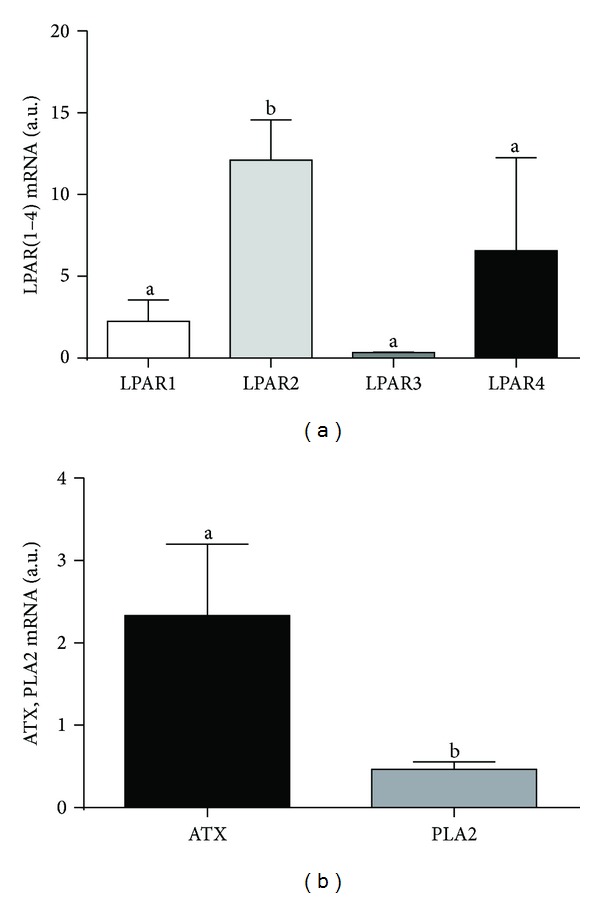
mRNA expression of (a) LPA receptors (*LPAR1–4*) and (b) autotaxin (*ATX*) and phospholipase A2 (*PLA2*) in oocytes. The values are expressed as mean ± SEM. Different letters indicate significant differences (*P* < 0.05), as determined by one-way ANOVA and Student's *t*-test, respectively.

**Figure 2:**
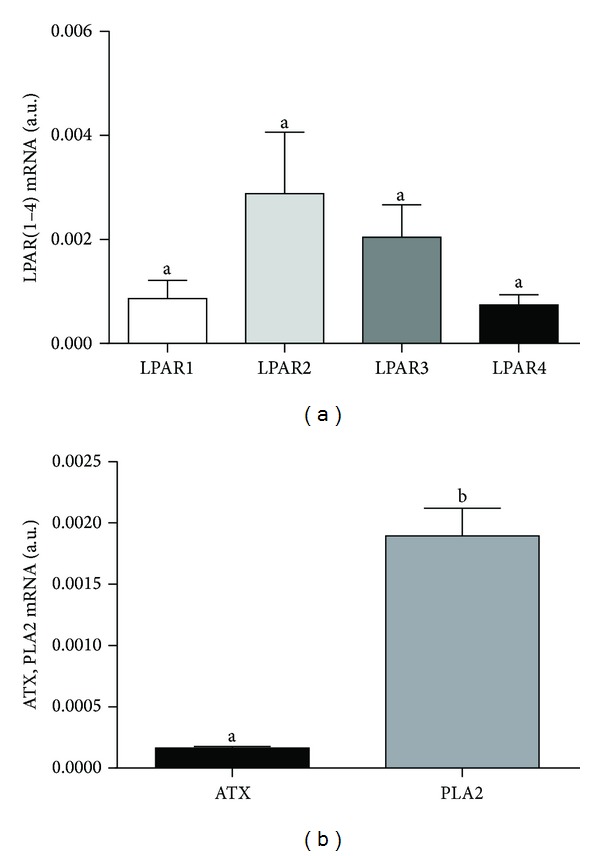
mRNA expression of (a) LPA receptors (*LPAR1–4*) and (b) autotaxin (*ATX*) and phospholipase A2 (*PLA2*) in cumulus cells. The values are expressed as mean ± SEM. Different letters indicate significant differences (*P* < 0.05), as determined by one-way ANOVA and Student's *t*-test, respectively.

After *in vitro* maturation of COCs, oocytes and cumulus cells transcribe genes coding for enzymes involved in LPA synthesis (*ATX* and *PLA2*) as well as *LPARs* (Figures 1 and 2). We found significantly higher mRNA expression of *LPAR2* than other three *LPARs* in bovine oocytes (Figure 1(a); *P* < 0.05). The expression of all examined *LPARs* in the cumulus cells did not significantly differ (Figure 2(a); *P* > 0.05). In the bovine oocytes the expression of *ATX* was higher than that of *PLA2* (Figure 1(b); *P* < 0.05), whereas in cumulus cells the opposite was observed (Figure 2(b); *P* < 0.05).

### 3.2. Effect of LPA on mRNA Abundance of Oocyte Quality Markers and Genes Involved in Apoptosis in Oocytes and Cumulus Cells after *In Vitro* Maturation

**Figure 3:**
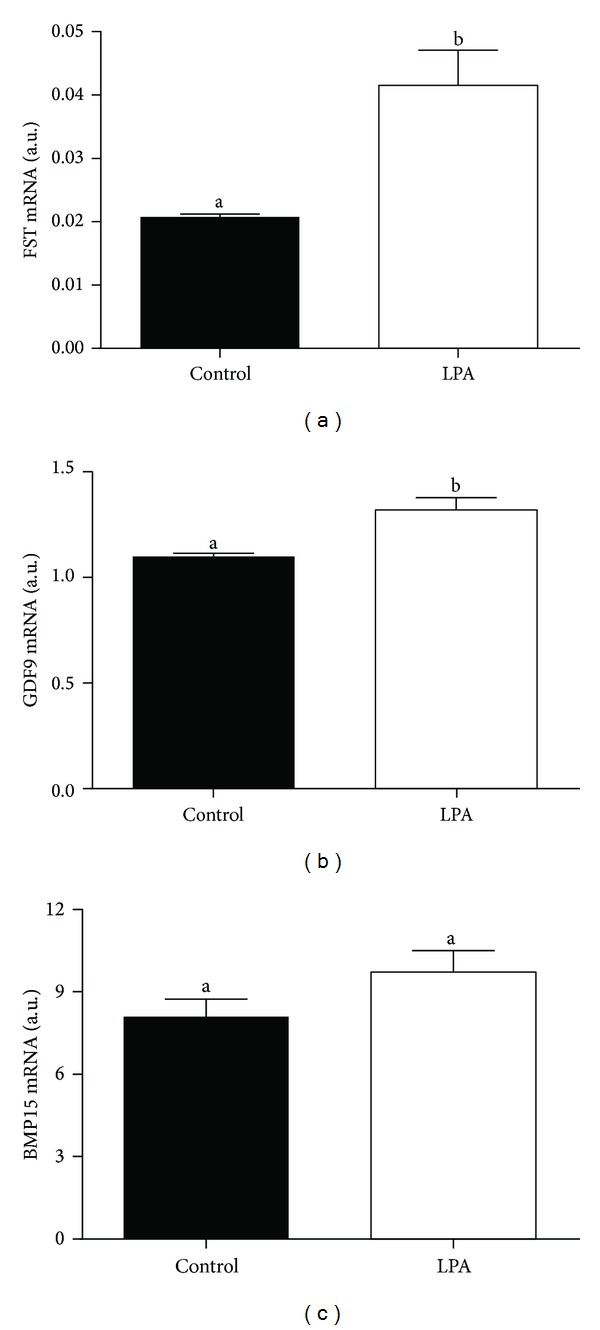
The effect of LPA (10^−5^ M) supplementation of maturation medium on mRNA abundance of *FST* (a), *GDF9* (b), and *BMP15* (c) in oocytes. The values are expressed as mean ± SEM. Different letters indicate significant differences (*P* < 0.05), as determined by Student's *t*-test.

**Figure 4:**
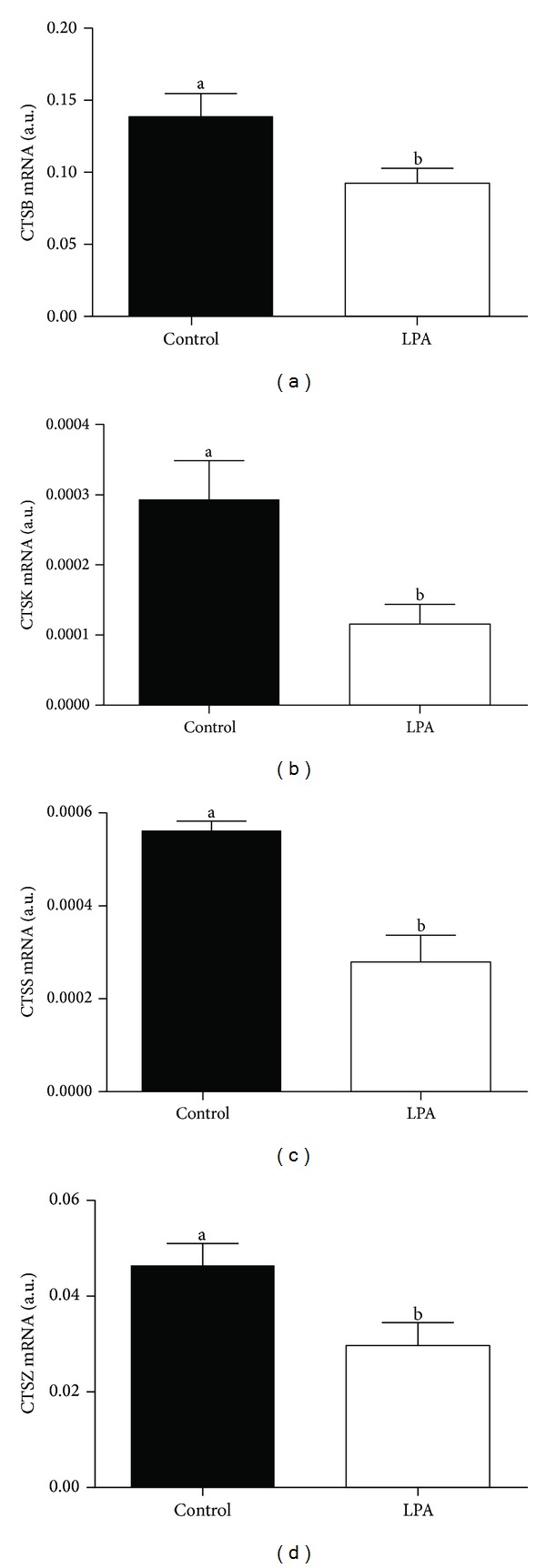
The effect of LPA (10^−5^ M) supplementation of maturation medium on mRNA abundance of *CTSB* (a), *CTSK* (b), *CTSS* (c), and *CTSZ* (d) in cumulus cells. The values are expressed as mean ± SEM. Different letters indicate significant differences (*P* < 0.05), as determined by Student's *t*-test.

**Figure 5:**
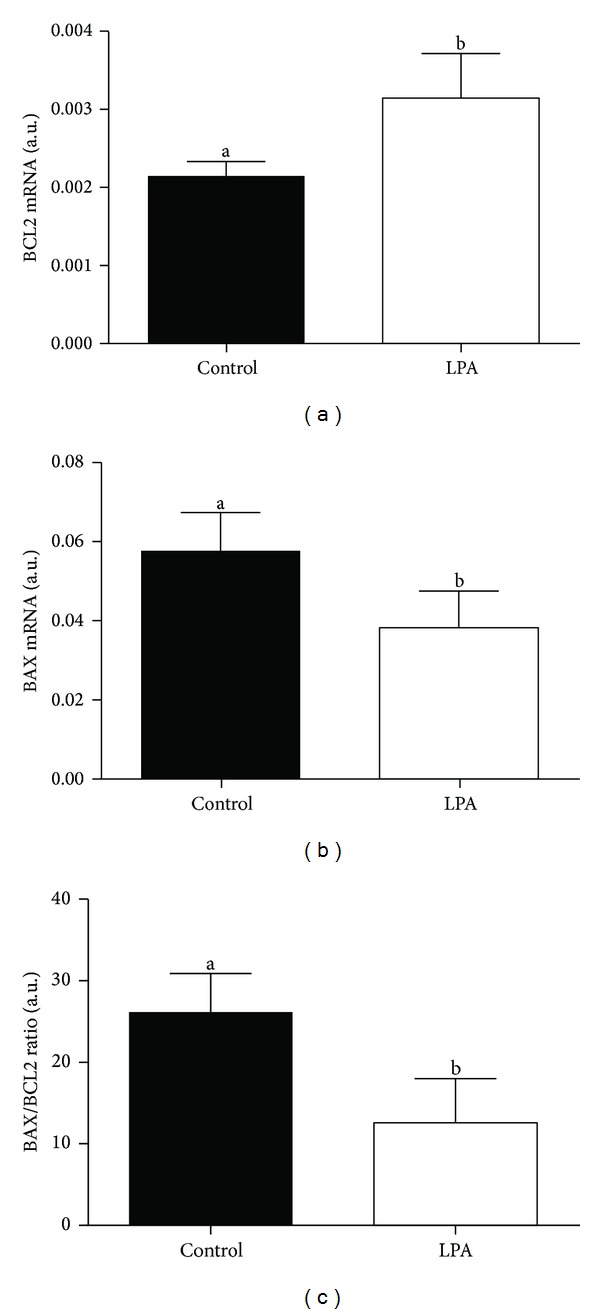
The effect of LPA (10^−5^ M) supplementation of maturation medium on mRNA abundance of *BCL2* (a), *BAX* (b), and *BAX/BCL2* ratio (c) in oocytes. The values are expressed as mean ± SEM. Different letters indicate significant differences (*P* < 0.05), as determined by Student's *t*-test.

**Figure 6:**
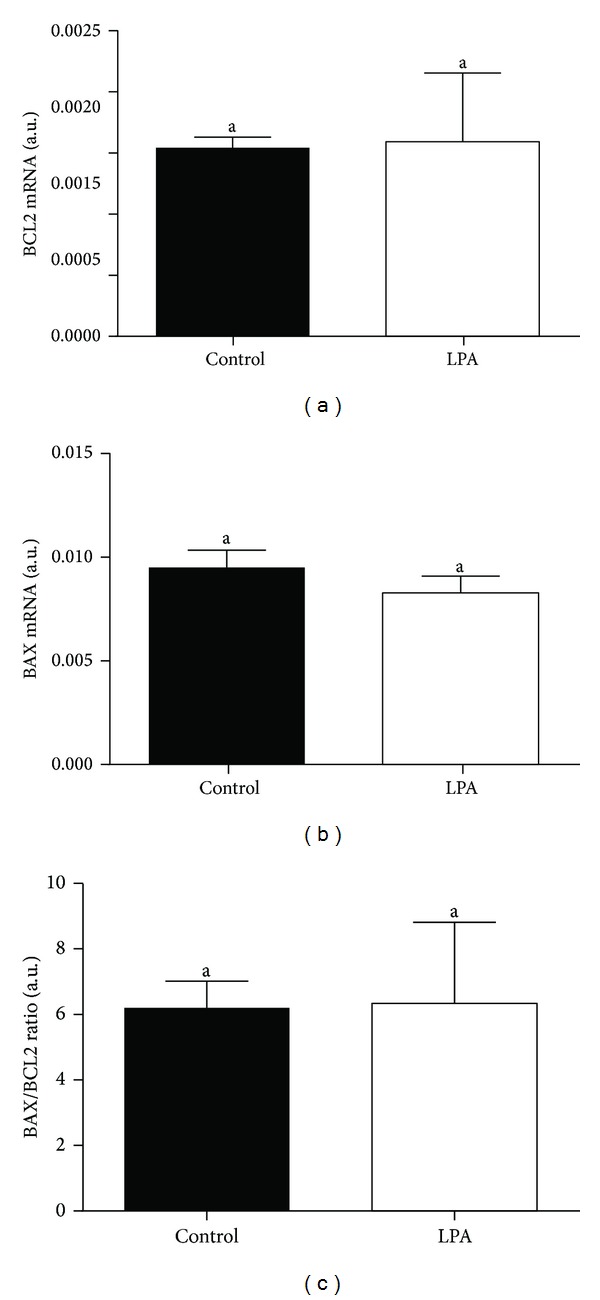
The effect of LPA (10^−5^ M) supplementation of maturation medium on mRNA abundance of *BCL2* (a), *BAX* (b), and *BAX/BCL2* ratio (c) in cumulus cells. The values are expressed as mean ± SEM. Different letters indicate significant differences (*P* < 0.05), as determined by Student's *t*-test.

We found higher mRNA abundance of *FST* and *GDF9* in the oocytes from the LPA-stimulated group compared to oocytes from the control group (Figures 3(a) and 3(b); *P* < 0.05). The supplementation of the maturation medium with LPA did not significantly influence *BMP15 *mRNA level in the examined oocytes (Figure 3(c); *P* > 0.05). In the cumulus cells there was lower mRNA abundance of all examined *CTSs* from the LPA-stimulated group compared to cumulus cells from the control group (Figure 4; *P* < 0.05). We demonstrated higher *BCL2* and lower *BAX* mRNA level in the oocytes from the LPA-stimulated group compared to oocytes from the control group (Figures 5(a) and 5(b); *P* < 0.05). The *BAX/BCL2* ratio was significantly lower in the oocytes matured in the presence of LPA compared to the oocytes from the control group (Figure 5(c); *P* < 0.05). The supplementation of the maturation medium with LPA did not significantly influence mRNA level of *BCL2* and *BAX *or *BAX/BCL2* ratio in the examined cumulus cells (Figure 6; *P* > 0.05).

### 3.3. Effect of LPA Supplementation of Maturation Medium on Embryonic Development

As shown in Table 2, we did not find any significant differences in the cleavage rates on day 2 between the control group and the LPA-stimulated group (61.7% versus 56.8%, resp.; *P* > 0.05). The blastocyst rates on day 7 were similar in the control group and the LPA-stimulated group (24.5% versus 28.4%, resp.; *P* > 0.05).

## 4. Discussion 

This study is the first to demonstrate mRNA expression of four types of *LPARs* and two main enzymes involved in LPA synthesis (*ATX* and *PLA2*) in bovine oocytes and cumulus cells. This indicates that bovine COCs are a potential source and target of LPA action and that LPA may be involved in cellular signaling between the oocyte and cumulus cells during maturation. Up to now, the presence of LPAR1 and LPAR2 was proposed only in the murine cumulus cells [35]. In mice it was also demonstrated that during blastocyst differentiation *in vitro*, embryos expressed *LPAR1* mRNA constitutively, *LPAR2* only in the late stage blastocysts, and there was no expression of *LPAR3* [38]. However, van Meeteren et al. [39] demonstrated mRNA expression of four LPA receptors during murine embryonic development *in vivo* from E6.5 to E10.5 with significantly higher expression of *LPAR1* than that of *LPAR2–4*. In ruminants, Liszewska et al. [17] showed that *LPAR1, LPAR2,* and *LPAR3* transcripts were expressed in ovine conceptuses during early pregnancy and postulated the main role of LPAR1 and LPAR3 at the time of implantation. In cows, we documented the presence of LPAR1 in the endometrium and four isoforms of LPARs in the CL with the dominant function of LPAR2 and LPAR4 [19, 20]. Moreover, in bovine granulosa cells four types of *LPARs* were expressed with the highest transcript abundance of *LPAR1 *[21].

The transcript level of *ATX* mRNA was only identified during early embryo development in mouse and it was shown that the offspring of ATX-knockout mice died during embryonic development [39]. In sheep, Liszewska et al. [17] detected expression of *ATX* in embryonic trophectoderm from day 12 to day 16 of pregnancy. In our previous study, we demonstrated the presence of ATX and PLA2 in stromal and epithelial cells of the bovine endometrium as well as mRNA expression of *ATX* and *PLA2* in granulosa cells [21, 40].

Considering that there is the possibility of LPA synthesis and action during *in vitro* maturation of COCs, in the second part of our study we examined the effect of LPA supplementation of maturation medium on mRNA abundance of the oocyte quality markers in oocytes and cumulus cells obtained after *in vitro* maturation. Supplementation of maturation medium with LPA increased oocyte *GDF9* and *FST* transcripts, whereas *BMP15* mRNA abundance was not affected. Morphological quality of oocytes can be depicted from the number and compactness of the neighboring cumulus cell layers [41]. Oocytes are responsible for cumulus cell expansion [42, 43], regulate steroid production [43–45], and maintain cumulus cell phenotype [45]. This is accomplished through the paracrine secretion of factors, which include TGF-*β* superfamily members, notably GDF9 and BMP15, also known as GDF-9B [46–48]. In cow, *BMP15* and *GDF9* transcription occur in oocyte during processes of *in vitro* maturation and fertilization and in preimplantation embryos until the five- to eight-cell or morula stage as well as in high quality oocytes [49–51]. Therefore, *BMP15* and *GDF9* are considered valid oocyte quality marker genes [50–52]. Moreover, Gendelman et al. [50] found that mRNA expression of *GDF9* was higher in early- versus late-cleaved embryos. Gendelman and Roth [51] documented higher *GDF9* transcript level in matured oocytes collected in the cold season than in those from the hot season and postulated that seasonally induced alterations in *GDF9* expression were involved in the reduced developmental competence noted for oocytes collected in the hot season. In fact, addition of BMP15 and GDF9 to maturation medium enhanced oocyte developmental competence in the cow [52]. Oocyte mRNA abundance of *FST* was associated with time of the first cleavage that accounted for high developmental competence of the oocyte [53]. Moreover, Lee et al. [54] showed higher level of FST protein in early versus late cleaving two-cell embryos and the stimulatory effects of FST on time to first cleavage, blastocyst rate, and cell allocation within the blastocyst. Here, increased transcription levels of *GDF9* and *FST* following LPA supplementation during *in vitro *maturation may indicate that LPA increased oocyte competence.

The role of cumulus cells during *in vitro* maturation is vital for oocyte maturation and subsequent fertilization and embryo development [55, 56]. Cumulus cells play a pivotal role in the provision of nutrients to the oocyte [57, 58] as well as stimulate oocyte glutathione synthesis [59]. Therefore, gene expression in cumulus cells may also reflect oocyte quality and competence. Bettegowda et al. [60] demonstrated negative correlation between *CTSs* transcript abundance in cumulus cells and oocyte quality as well as their developmental competence. Here, LPA supplementation of maturation medium decreased cumulus cell transcript levels of *CTSB*, *CTSK*, *CTSS,* and *CTSZ*. Again, this may indicate that LPA increased oocyte competence.

LPA supplementation of maturation medium had no effect on transcription levels of proapoptotic (*BAX*) and antiapoptotic (*BCL2*) genes in cumulus cells but decreased oocyte *BAX* mRNA abundance and increased oocyte *BCL2* transcript levels. Moreover, the *BAX/BCL2* ratio was lower in the oocytes matured in the presence of LPA compared to the oocytes from the control group. Apoptosis in cumulus cells may be also a good marker of oocyte developmental competence [61] due to the bidirectional communication between oocytes and cumulus cells [62]. Cumulus cells regulate nuclear and *cytoplasmic *maturation of oocytes and prevent apoptosis induced by oxidative stress during *in vitro* maturation [63, 64]. However, the relationship between the occurrence of apoptosis in cumulus cells and oocyte developmental competence is controversial [65–69]. In fact, some studies report that oocytes with early signs of atresia are developmentally more competent [70, 71]. Oocytes with the highest transcriptional level of *BAX* and the lowest mRNA level of *BCL2* exhibited the highest nuclear maturation, cleavage, and blastocyst rate [71]. In contrast, in other studies, good quality oocytes showed the highest transcription levels of *BCL2* and the lowest mRNA abundance of *BAX* [72]. The ratio of *BCL2* to *BAX* may be an indicator of the tendency of oocytes and embryos towards either survival or apoptosis [72]. According to Yuan et al. [73] COCs with no signs of atresia yield higher blastocyst rates. The authors found that the degree of apoptosis in the cumulus cells is negatively correlated to the developmental competence of oocyte [73]. On the other hand, another group of authors demonstrated that the level of apoptosis in cumulus cells does not correlate either with COC morphology or oocyte meiotic stage [74]. Here, LPA decreased *BAX/BCL2* ratio, indicating that an antiapoptotic balance was induced in the oocyte, which may be relevant for oocyte competence. Hussein et al. [75] showed that, at the beginning, the apoptotic signal appears in the cumulus cells and then in the oocyte. Similarly, we have detected an antiapoptotic effect of LPA in cultured luteal cells: LPA inhibited the stimulatory effects of tumor necrosis factor alpha (TNF*α*) and interferon gamma (IFN*γ*) on the expression of BAX mRNA and protein in steroidogenic luteal cells [76].

Although, LPA supplementation of maturation medium promoted transcription of quality marker genes in oocytes, decreased transcription of *CTSs* in cumulus cells, and induced and antiapoptotic balance in oocytes, this was not translated into a higher number of cleaved embryos and subsequent blastocyst development. Ye et al. [77] examined the influence of LPA on the embryo implantation in LPA3 receptor null mice. According to these authors, examined mice exhibited delayed implantation, reduced number of implantation sites, delayed embryonic development, and increased embryonic mortality [77]. However, we did not examine the effect of LPA on the implantation of bovine embryos. Moreover, we cannot exclude that LPA supplementation of maturation medium can impact the maturation process itself and/or early pronuclear stages of embryo development. In rodents, LPA promoted nuclear and cytoplasmic oocyte maturation *via* cumulus cells and through the closure or loosening of gap junctions between cumulus cells and the oocyte [35, 36], as well as stimulated blastocyst development [38, 78, 79]. Differences in early embryonic development between the mouse and bovine models may account for the discrepancy in the rate of blastocyst development observed in our study and in the studies with rodents. Further studies are needed to evaluate the role of LPA stimulation during *in vitro* maturation and embryo culture in *in vivo* survival of bovine embryos.

In conclusion, our study demonstrates for the first time that bovine COCs (both oocytes and cumulus cells) are a potential source and target of LPA action. We postulate that LPA exerts an autocrine and/or paracrine signaling, through several LPARs, between the oocyte and cumulus cells. LPA supplementation of maturation medium increases oocyte transcripts of quality marker genes (*FST* and *GDF9*), promotes an antiapoptotic balance in transcription of genes involved in apoptosis (*BCL2 *and *BAX*), and decreases cumulus cells transcripts associated with low viability (*CTSs*). These effects, although not affecting *in vitro* development until the blastocyst stage, may be of relevance for subsequent *in vivo* developmental competence.

## Figures and Tables

**Table 1:** Primers used for real-time PCR.

Gene	Primer sequence (5′-3′)	Fragment size, bp	GenBank accession number
*LPAR1 *	ACGGAATCGGGATACCATGA	86	NM_174047.2
	CCAGTCCAGGAGTCCAGCAG		

*LPAR2 *	TTCTATGTGAGGCGGCGAGT	161	NM_001192235.1
	AGACCATCCAGGAGCAGCAC		

*LPAR3 *	TCCAACCTCATGGCCTTCTT	101	NM_001192741.2
	GACCCACTCGTATGCGGAGA		

*LPAR4 *	CCACAGTACCTCCAGAAAGTTCA	192	NM_001098105.1
	TTGGAATTGGAAGTCAATGAATC		

*ATX *	ACCCCCTGATTGTCGATGTG	120	NM_001080293.1
	TCTCCGCATCTGTCCTTGGT		

*PLA2 *	CTGCGTGCCACAAAAGTGAC	92	NM_001075864.1
	TCGGGGGTTGAAGAGATGAA		

*FST *	GCAGCTCTACATGCGTGGTG	133	NM_175801.2
	TGACAGGCACTGGGGTAGGT		

*GDF9 *	TCGGACATCGGTATGGCTCT	86	NM_174681.2
	GGATGGTCTTGGCACTGAGG		

*BMP15 *	GCAGAGGAAGCCTCGGATCT	104	NM_001031752.1
	CAATGGTGCGGTTTTCCCTA		

*CTSB *	GGCTCACCCTCTCCAGTCCT	136	NM_174031.2
	TCACAACCGCCTTGTCTGAA		

*CTSK *	GAACCACTTGGGGGACATGA	77	NM_001034435.1
	GGGAACGAGAAGCGGGTACT		

*CTSS *	CCGCCGTCAGCATTCTTAGT	99	NM_001033615.1
	CATGTGCCATTGCAGAGGAG		

*CTSZ *	GGGGAGGGAGAAGATGATGG	146	NM_001077835.1
	CCACGGAGACGATGTGGTTT		

*BCL2 *	GAGTTCGGAGGGGTCATGTG	203	NM_001166486.1
	GCCTTCAGAGACAGCCAGGA		

*BAX *	GTGCCCGAGTTGATCAGGAC	126	NM_173894.1
	CCATGTGGGTGTCCCAAAGT		

*GAPDH *	CACCCTCAAGATTGTCAGCA	103	NM_001034034.2
	GGTCATAAGTCCCTCCACGA		

**Table 2:** The effect of LPA supplementation of *in vitro* maturation medium on cleavage and blastocyst rates on day 2 and day 7, respectively.

Supplement	Matured oocytes, *n*	Cleaved embryos, *n*	Cleavage rate, %	Blastocyst on day 7, *n*	Blastocyst rate, %
Control (PBS)	188	116	61,7	46	24,5
LPA (10^−5^ M)	190	108	56,8	54	28,4

Proportion of cleaved embryos and of blastocysts relative to the total number of matured oocytes.
